# Investigation on integro-differential equations with fractional boundary conditions by Atangana-Baleanu-Caputo derivative

**DOI:** 10.1371/journal.pone.0301338

**Published:** 2024-05-31

**Authors:** Samy A. Harisa, Nashat Faried, V. Vijayaraj, C. Ravichandran, Ahmed Morsy

**Affiliations:** 1 Department of Mathematics, College of Arts and Sciences, Prince Sattam bin Abdulaziz University, Wadi Aldawaser, Saudi Arabia; 2 Department of Mathematics, Faculty of Science, Ain Shams University, Cairo, Egypt; 3 Department of Mathematics, Kongunadu Arts and Science College, Coimbatore, Tamil Nadu, India; Qujing Normal University, CHINA

## Abstract

We establish, the existence and uniqueness of solutions to a class of Atangana-Baleanu (AB) derivative-based nonlinear fractional integro-differential equations with fractional boundary conditions by using special type of operators over general Banach and Hilbert spaces with bounded approximation numbers. The Leray-Schauder alternative theorem guarantees the existence solution and the Banach contraction principle is used to derive uniqueness solutions. Furthermore, we present an implicit numerical scheme based on the trapezoidal method for obtaining the numerical approximation to the solution. To illustrate our analytical and numerical findings, an example is provided and concluded in the final section.

## 1 Introduction

Fractional differential equations (FDEs) seemed as an excellent mathematical tool for modeling of many physical phenomena appearing in various branches, such as viscoelasticity, self-similar protein dynamics, continuum and statistical mechanics, dynamics of particles etc [1, 2]. A number of non-integer derivatives and their associated integrals have been developed and a systematic classification of fractional integrals with certain generalized Leibniz (type) rules was presented in [3]. Consequently, fractional calculus allows us to choose what kind of derivative to be used to solve a model, resulting in more accurate solutions [4–7].

Recently many authors have studied mathematical models involving AB fractional derivative. AB derivative in Caputo sense has also received considerable attention from researchers in the theoretical area of nonlinear FDEs [8, 9]. For instance, [10–12] addressed the existence of solutions to nonlinear Cauchy problems containing AB derivative.

Researchers in the theoretical domain of nonlinear fractional differential equations have shown significant interest in the Caputo sense AB derivative as well [13–16]. In this situation, a fractional derivative with a nonsingular kernel that relied on the M-L function was introduced by Atangana and Baleanu(A-B). This new dimension demonstrates the close connection between fractional calculus and the M-L function. It helps us to more effectively handle computing needs, nonlocal dynamics, and distinguish between various traits. [17–19].

In addition, the Gronwall inequality was established in the context of AB integral in order to analyze the Ulam-Hyers stability of the solution [20, 21]. The existence and qualitative behaviors of solutions of fractional integro-differential equations(FIDE) were investigated and which involved an extension model of [15, 22–24]. In [26], the authors proposed a new explicit numerical scheme based on two-step Lagrange polynomial for solving nonlinear FDEs of AB derivative and also presented the error analysis.

[25–28] has defined higher-order AB fractional operators and established some useful relations among the operators. Some recent progress on the existence of solutions to various higher-order nonlinear FDEs based on AB derivative with classical boundary conditions can be found in a series of papers [29–37].

Now, we consider the nonlinear FIDE with fractional boundary conditions of the following from:

ABCDa+qw(t)=F(t,∫0tK(t,s)w(s)ds,∫0tH(t,s)w(s)ds),1<q<2,t∈[0,a]=J, (1.1)

w(0)=w0,ABCDa+q-1w(a)=w1. (1.2)

Where ABCDa+q and ABCDa+q−1 denote the AB fractional derivatives (FD) in Caputo sense; ℱ is any real-valued absolutely continuous function and *w*_0_, *w*_1_ are real numbers. Here, K,ℋ are given function which fulfills certain conditions to be defined later on.

For our convenient, we consider $Sw(t)=\int_{0}^{t}K(t,s)w(s)ds$ and $Tw(t)=\int_{0}^{t}ℋ(t,s)w(s)ds$.

Then (1.1) becomes,

{ABCDa+qw(t)=F(t,Sw(t),Tw(t)),1<q<2,t∈[0,a]=J,w(0)=w0,ABCDa+q-1w(a)=w1. (1.3)

In this work, we derive the necessary preliminaries in Section 2. In Section 3, some basic properties of AB operators, along with an auxiliary lemma for the linear version of (1.1), are derived. In Section 4, we study the existence of solution to (1.1). Section 5 contains an implicit numerical scheme for solving the proposed nonlinear FDE (1.1). In Section 6, illustrative examples for our obtained results are provided with their numerical simulations.

## 2 Preliminaries

Here, we recall basic concepts and useful lemmas related to AB fractional derivatives and AB fractional integral. Let *C*[*a*, *b*], *a* < *b*, be the Banach space of all real-valued continuous functions on [*a*, *b*] with the norm ||*y*||_∞_ = *sup*_*t*∈[*a*,*b*]_{|*y*(*t*)|}. Let *AC*[*a*, *b*] be the space of all real value absolutely continuous functions on [*ab*] and

$$\begin{array}{c} AC^{m}\left[a,b\right]=\left\{y|y:\left[a,b\right]\to R,y^{\left(m-1\right)}\in AC\left[a,b\right]\right\},m\in N. \end{array}$$

**Definition 2.1** [19, 26] *The classical & two-parametric Mittag-Leffler functions are*,

$$\begin{array}{c} E_{p}\left(z\right)=\sum_{k=0}^{\infty }\frac{z^{k}}{\Gamma (kp+1)},E_{p,q}\left(z\right)=\sum_{k=0}^{\infty }\frac{z^{k}}{\Gamma (kp+q)},\left(z,p,q\in C,R\left(p\right)>0\right), \end{array}$$

respectively, where *E*_*p*,1_(*z*) = *E*_*p*_(*z*).

**Definition 2.2** [2, 26, 38] *The RL fractional integral of order q* > 0 *is*, $\left(I_{a^{+}}^{q}w\right)(t)=\frac{1}{\Gamma (q)}\int_{a}^{t}(t-r{)}^{q-1}w(r)dr,$ *where w* ∈ *L*^1^(*a*, *b*).

**Remark 2.3** [26] *For* 0 < *q* < 1, $I_{a^{+}}^{q}$ *maps AC*[*a*, *b*] *into AC*[*a*, *b*] *and maps C*[*a*, *b*] *into C*[*a*, *b*].

**Definition 2.4** [8, 10, 18, 26, 32] *Let* 0 < *q* < 1. *The AB FD in Caputo sense is*,

(ABCDa+qw)(t)=B(q)(1-q)∫atEq[-q1-q(t-r)q]w′(r)dr,

*where w* ∈ *AC*[*a*, *b*].

*The AB FD in RL sense is*,

(ABRDa+qw)(t)=B(q)(1-q)ddt∫atEq[-q1-q(t-r)q]w(r)dr,

*where w* ∈ *L*^1^(*a*, *b*).

*The AB fractional integral is*,

(ABIa+qw)(t)=1-qB(q)w(t)+qB(q)(Ia+qw)(t),

*where w* ∈ *L*^1^(*a*, *b*). *Now, we consider the normalization function* ℬ(q) *to be real-valued strictly positive such that* ℬ(0)=ℬ(1)=1.

**Lemma 2.5** [8, 26] *Let* 0 < *q* < 1. *Then*

(ABCDa+qw)(t)=(ABRDa+q{F(Sw(t),Tw(t))})-B(q)(1-q)w(a)Eq[-q1-q(t)q], (2.1)

*where w* ∈ *AC*[*a*, *b*].

The higher-order AB fractional derivatives and integral defined in [3, 26, 29–37] based on Definition 2.4 are as follow:

**Definition 2.6** [3, 26] *Let m* < *q* < *m* + 1(*m* ∈ *N*_0_) *and p* = *q* − *m and N*_0_ *is denoting* 0.

*The AB FD in caputo sense is*,

(ABCDa+qw)(t)=(ABCDa+q{F(t,Sw(t),Tw(t))}),

*where w* ∈ *AC*^*m*+1^[*a*, *b*].

*The AB FD in RL sense is*,

(ABRDa+qw)(t)=(ABRDa+q{F(t,Sw(t),Tw(t))}),

*where w* ∈ *L*^1^(*a*, *b*).

*The AB fractional integral is*,

(ABIa+qw)(t)=(Ia+mIa+p{F(t,Sw(t),Tw(t))}),

*where w* ∈ *L*^1^(*a*, *b*).

**Lemma 2.7** [3, 26] *Let m* < *q* < *m* + 1(*m* ∈ *N*_0_). *Then*

(ABIa+qABCDa+qw)(t)={F(t,Sw(t),Tw(t))}-∑k=0mw(k)(a)k!(t-k)k, (2.2)

*where w* ∈ *AC*^*m*+1^[*a*, *b*].

## 3 Equivalent linear system solutions

This section is devoted to obtaining several important properties and results that will be helpful in our forthcoming discussion. We first define the Laplace transform of higher-order AB operators as follows:

**Proposition 3.1.** [26] Let $m<q<m+1\left(m\in {\mathbb{N}}_{0}\right)$. Then, in particular *a* = 0, we get

L{(ABCD0+qw)(t)}(s)=B(q-m)m+1-psqL{F(t,Sw(t),Tw(t))}(s)-∑0≤k≤msq-k-1w(k)(0)sq-m+q-mm+1-p,L{(ABRD0+qw)(t)}(s)=B(q-m)m+1-psqL{F(t,Sw(t),Tw(t))}(s)-∑0≤k≤m-1sq-k-1w(k)(0)sq-m+q-mm+1-p,L{(ABI0+qw)(t)}(s)=1B(q-m)[(m+1-p)s-m+(q-m)s-q]L{F(t,Sw(t),Tw(t))}(s),

provided the Laplace transform of *w* exists.

**Lemma 3.2** [26] Let *m* < *q* < *m* + 1(*m* ∈ *N*_0_). Then, in particular *a* = 0 yields the following result,

(ABCDa+qw)(t)=(ABRDa+q{F(t,Sw(t),Tw(t))})-B(q-m)m+1-pw(m)Eq-m[-q-mm+1-p(t)q-m],

where *w* ∈ *AC*^*m*+1^[*a*, *b*].

**Proposition 3.3** [26] Let *m* < *q* < *m* + 1(*m* ∈ *N*_0_). Then,

(ABIa+qABCDa+qw)(t)={F(t,Sw(t),Tw(t))}-w(a)Eq-m[-q-mm+1-p(t)q-m],

where *w* ∈ *AC*[*a*, *b*].

**Proof:** From [26] and Definition 2.4, by setting *p* = *q* − *m* and using AB fractional derivative in Caputo sense and AB fractional integral operators, we get

(ABIa+qABCDa+qw)(t)=(ABIa+qdmdtmABCDa+q{F(t,Sw(t),Tw(t))})=(ABIa+qABCDa+q{F(t,Sw(t),Tw(t))}).

Hence the final conclusion follows from Eq 2.1 and [[3], Proposition 3.1].

**Lemma 3.4** [26] Let 0 < *α* ≤ 1 and λ > 0. Then, for all *x* > 0, the function *xE*_*α*,2_(−*λx*^*α*^) is strictly positive and monotonically increasing with respect to ‘x’.

**Proof.** From [2, 26], we have

$$\begin{array}{cc} & \int_{0}^{x}t^{\beta -1}E_{\alpha ,\beta }\left(-\lambda t^{\alpha }\right)dt=x^{\beta }E_{\alpha ,\beta +1}\left(-\lambda x^{\alpha }\right),\beta >0. \end{array}$$

In particular, by setting *β* = 1, the above equation reduces to

$$\begin{array}{cc} & xE_{\alpha ,2}\left(-\lambda x^{\alpha }\right)=\in_{0}^{x}E_{\alpha }\left(-\lambda t^{\alpha }\right)dt. \end{array}$$

From[[39] in Lemma 2.2], *xE*_*α*,2_(−*λx*^*α*^), *x* > 0, is strictly positive and monotonically increasing with respect to ‘x’.

**Remark 3.5** [26] Let, $\lambda =\frac{q-1}{2-q}$, 1 < *q* < 2, from Lemma 3.4, for all *x* > 0,

$$\begin{array}{cc} & xE_{q^{-}1,2}[-\frac{q-1}{2-q}x^{q-1}]>0. \end{array}$$

**Lemma 3.6** Let ℱ∈AC[0,b] with ℱ(0)=0 and 1 < *q* < 2. Then the solution to the linear system

{ABCDa+qw(t)=F(t,Sw(t),Tw(t)),t∈[0,a]w(0)=w0,ABCDa+q-1w(b)=w1 (3.1)

is

$$\begin{array}{ccc} & w\left(t\right)=w_{0}+\frac{1}{\Delta }[w_{1}-\int_{0}^{b}F\left(r,Sw\left(r\right),Tw\left(r\right)\right)dr]t+\frac{2-q}{B(q-1)}F\left(r,Sw\left(r\right),Tw\left(r\right)\right)dr & \\ & \;+\frac{1}{B(q-1)\Gamma (q-1)}\int_{0}^{t}{\left(t-r\right)}^{q-1}F\left(r,Sw\left(r\right),Tw\left(r\right)\right)dr,\text{for}\;\text{all}\;\;t\in J, \end{array}$$ (3.2)

where,

$$\begin{array}{cc} & \Delta =\frac{B(q-1)}{2-q}bE_{p-1,2}[-\frac{q-1}{2-q}b^{q-1}]. \end{array}$$

**Proof.** Use of Atangana-Baleanu integral operator ABI0+q on both sides of the equation in (3.1) with the help of Eq 2.2, results in

w(t)=c1+c2t+ABI0+qF(t,Sw(t),Tw(t))=c1+c2t+2-qB(q-1)∫0tF(r,Sw(r),Tw(r))dr+1B(q-1)Γ(q-1)∫0t(t-r)q-1F(r,Sw(r),Tw(r))dr, (3.3)

where $c_{1},c_{2}\in \mathbb{R}$. The condition *w*(0) = *w*_0_ yields *c*_1_ = *w*_0_. Moreover, by Definition 2.6, we have

w(t)=w0+c2t+I0+ABI0+q-1F(t,Sw(t)Φ,Tw(t)).

In view of Proposition 3.3, we obtain

ABCD0+q-1w(t)=c2B(q-1)2-qtEp-1,2[-q-12-qtq-1]+∫0tF(r,Sw(r),Tw(r))dr.

By using boundary condition at *t* = *b* given in (3.1), it follows that

$$\begin{array}{c} c_{2}=\frac{1}{\Delta }[w_{1}-\int_{0}^{b}F\left(r,Sw\left(r\right),Tw\left(r\right)\right)dr]. \end{array}$$

Substitution of the above values of *c*_1_ and *c*_2_ into (3.3) leads to the desired solution given in (3.2). This Lemma derive the integral equation for the problem (1.1).

**Remark 3.7** [26] From 3.2, one can obtain

$$\begin{array}{ccc} & w^{\prime }\left(t\right)=\frac{1}{\Delta }[w_{1}-\int_{0}^{b}F\left(r,Sw\left(r\right),Tw\left(r\right)\right)dr]t+\frac{2-q}{B(q-1)}\left\{F\left(r,Sw\left(r\right),Tw\left(r\right)\right)dr\right\} & \\ & \;+\frac{1}{B(q-1)\Gamma (q-1)}\int_{0}^{t}{\left(t-r\right)}^{q-1}F\left(r,Sw\left(r\right),Tw\left(r\right)\right)dr. \end{array}$$

Therefore, our assumption ℱ∈AC(J) together with Remark 2.3, that *y* ∈ *AC*^2^(*J*). By Definition 2.6, we can define the AB fractional derivative of the function *y* in Caputo sense on *J*. Moreover, it follows from Proposition 3.3 that *w* satisfies the linear problem (3.1).

For the elementary results of the Eq (1.3), we need some following hypotheses:

(H1) $\left|ℱ(r,(Sw(r),Tw(r)))|\leq \varphi_{1}(t)+\varphi_{2}(t)|w\right|$, for all (*t*, *w*) ∈ Ω and $ℱ\subseteq {ℛ}^{3}$.
(H2) $\left|ℱ(t,w)-ℱ(t,\tilde{w})|\leq \phi (t)|w-\tilde{w}\right|$ for a.e. $(t,w),(t,\tilde{w})\in \Omega$ and $ℱ\subseteq {ℛ}^{3}$.
(H3) Let *w* ∈ *C*[0, *T*] & function φ∈(C[0,T]×ℛ×ℛ×ℛ,ℛ) is continuous function & a positive constant *ζ*_1_, *ζ*_2_ & *ζ* s.t., $\|\varphi (J,w_{1},w_{2},w_{3})-\varphi (J,\varphi_{1},\varphi_{2},\varphi_{3})\|\leq \zeta_{1}\left(\left\|w_{1}-\varphi_{1}\right\|+\left\|w_{2}-\varphi_{2}\right\|+\left\|w_{3}-\varphi_{3}\right\|\right)\forall$ *w*_1_, *w*_2_, *w*_3_, *φ*_1_, *φ*_2_, *φ*_3_ in *w*. $\zeta_{2}=max_{w\in R}\|f(,R,R,R)\|\&\zeta =max\{\zeta_{1},\zeta_{2}\}$.

## 4 Existence result

We first convert the FIDE (1.1) to an integral equation and then consider a fixed-points problem for an integral operator. On the basis of Lemma 3.6, and solution operator G:C(T)→C(T) as follows:

$$\begin{array}{ccc} & \left(Gw\right)\left(t\right)=w_{0}+\frac{1}{\Delta }[w_{1}-\int_{0}^{b}F\left(r,\left(Sw\left(r\right),Tw\left(r\right)\right)\right)dr]t & \\ & \;+\frac{2-q}{B(q-1)}\int_{0}^{t}F\left(r,\left(Sw\left(r\right),Tw\left(r\right)\right)\right)dr & \\ & \;+\frac{1}{B(q-1)\Gamma (q-1)}\int_{0}^{t}{\left(t-r\right)}^{q-1}F\left(r,\left(Sw\left(r\right),Tw\left(r\right)\right)\right)dr,\;\;\text{for}\;\text{all}\;t\in J. \end{array}$$ (4.1)

We now only need to seek the fixed-points of G in C(T), because the fixed-points of G are solutions of (1.1). For brevity, we use the notations

$$\begin{array}{ccc} & \Lambda =\frac{b}{\Delta }+\frac{2-q}{B(q-1)}+\frac{b^{q-1}}{B(q-1)\Gamma (q-1)} & \\ & \tilde{\Lambda }=\frac{b^{2}}{\Delta }+\frac{b(2-q)}{B(q-1)}+\frac{b^{q}}{qB(q-1)\Gamma (q-1)}. \end{array}$$

By considering the growth condition on nonlinearity, we now establish the result based on Leray-Schauder alternative theorem [40].

**Theorem 4.1** *Let* $\Omega =\{(t,w)\in {ℛ}^{2}:t\in J$ *and* |*w*| ≤ λ *for fixed* λ > 0}. *Suppose that* ℱ:Ω→ℛ *is absolutely continuous and there exist φ*_*i*_ ∈ *C*(*J*, [0, ∞)), *i* = 1, 2 and if ℱ(0,w(0))=0, *then* (1.1) *has a solution in C*(*T*), *provided*

$$\begin{array}{c} \Lambda \int_{0}^{b}\varphi_{2}(F(r,(Sw(r),Tw(r))))dr<1. \end{array}$$

***Proof*.** *The operator* G:C(T)→C(T) *is completely continuous*.

*Suppose* D⊂C(T) *is an arbitrary bounded set. Then, by using (H1), there is a number RL* > 0, *such that* |ℱ(r,(Sw(r),Tw(r)))|≤RL for t∈T,w∈D.

*Now*, ∀w∈D, *we obtain*

$$\begin{array}{ccc} & \left|\left(Gw\right)\left(t\right)\right|\leq |w_{0}|+\frac{1}{\Delta }[|w_{1}|+\int_{0}^{b}\left|F\left(r,\left(Sw\left(r\right),Tw\left(r\right)\right)\right)\right|dr]t & \\ & \;+\frac{2-q}{B(q-1)}\int_{0}^{t}\left|F\left(r,\left(Sw\left(r\right),Tw\left(r\right)\right)\right)\right|dr & \\ & \;+\frac{1}{B(q-1)\Gamma (q-1)}\int_{0}^{t}{\left(t-r\right)}^{q-1}\left|F\left(r,\left(Sw\left(r\right),Tw\left(r\right)\right)\right)\right|dr & \\ & \;\leq |w_{0}|+\frac{1}{\Delta }[|w_{1}|+\frac{b}{\Delta }+\frac{2-q}{B(q-1)}+\frac{b^{q-1}}{B(q-1)\Gamma (q-1)}+\frac{b^{2}}{\Delta }+\frac{b(2-q)}{B(q-1)} & \\ & \;+\frac{b^{q}}{q^{B}\left(q-1\right)\Gamma \left(q-1\right)}\left|F\left(r,\left(Sw\left(r\right),Tw\left(r\right)\right)\right)\right|dr] & \\ & \;\leq |w_{0}|+\frac{b}{\Delta }|w_{1}|+RL\tilde{\Lambda }<+\infty , \end{array}$$

⇒G(D) *is bounded*.

*Next, we show that* G(D) *is equicontinuous. For all* w∈D *and* 0 ≤ *τ*_1_ < *τ*_2_ ≤ *b*, *we have*

$$\begin{array}{ccc} & \left|\left(Gw\right)(\tau_{2})-\left(Gw\right)(\tau_{1})\right| & \\ & \leq \frac{1}{\Delta }[|w_{1}|+\int_{0}^{b}|F\left(r,\left(Sw\left(r\right),Tw\left(r\right)\right)\right)dr](\tau_{2}-\tau_{1}) & \\ & \;+\frac{2-q}{B(q-1)}\int_{\tau_{1}}^{\tau^{\tau_{2}}}\left|F\left(r,\left(Sw\left(r\right),Tw\left(r\right)\right)\right)\right|dr & \\ & \;+\frac{1}{B(q-1)\Gamma (q-1)}[\int_{0}^{\tau_{1}}[{\left(\tau_{2}-r\right)}^{q-1}-{\left(\tau_{1}-r\right)}^{q-1}]\left|F\left(r,\left(Sw\left(r\right),Tw\left(r\right)\right)\right)\right|dr & \\ & \;+\int_{\tau_{1}}^{\tau_{2}}{\left(\tau_{2}-r\right)}^{q-1}\left|F\left(r,\left(Sw\left(r\right),Tw\left(r\right)\right)\right)\right|dr] & \\ & \leq [\frac{|w_{1}|+bRL}{\Delta }+\frac{2-q}{B(q-1)}RL](\tau_{2}-\tau_{1})+\frac{RL}{qB(q-1)\Gamma (q+1)}(\tau_{2}^{q}-\tau_{1}^{q}). \end{array}$$

*Since the function τ*^*q*^, 1 < *q* < 2, *is uniformly continuous on* J,G(D) *is a family of equicontinuous functions. Therefore, following the Arzela-Ascoli theorem*, G(D) is relatively compact set in *C*(*T*). *Hence, we conclude that* G *is completely continuous*.

*Now, we prove* S={w∈C(T):w=λGw *and* λ ∈ (0, 1)} *is a bounded set*.

*Let* w∈S , *then* w(t)=λ(Gw)(t) *for t* ∈ *T*. *By (H1), for t* ∈ *T*, *one has*

$$\begin{array}{ccc} & \left|w(t)|=|\lambda (G,F(r,(Sw(r),Tw(r)))\right| & \\ & \;<|w_{0}|+\frac{1}{\Delta }[|w_{1}|+\int_{0}^{b}(\varphi_{1}\left(F\left(r,\left(Sw\left(r\right),Tw\left(r\right)\right)\right)\right)+\varphi_{2}\left(r\right)\left|F\left(r,\left(Sw\left(r\right),Tw\left(r\right)\right)\right)\right|)dr]t & \\ & \;+\frac{2-q}{B(q-1)}\int_{0}^{t}(\varphi_{1}\left(r\right)+\varphi_{2}\left(r\right)\left|F\left(r,\left(Sw\left(r\right),Tw\left(r\right)\right)\right)\right|)dr & \\ & \;+\frac{1}{B(q-1)\Gamma (q-1)}\int_{0}^{t}{\left(t-r\right)}^{q-1}(\varphi_{1}\left(r\right)+\varphi_{2}\left(r\right)\left|F\left(r,\left(Sw\left(r\right),Tw\left(r\right)\right)\right)\right|)dr & \\ & \;\leq |w_{0}|+\frac{1}{\Delta }[|w_{1}|+\frac{b}{\Delta }+\frac{2-q}{B(q-1)}+\frac{b^{q-1}}{B(q-1)\Gamma (q-1)}\int_{0}^{b}\varphi_{1}\left(r\right)dr+\frac{b}{\Delta }+\frac{\left(2-q\right)}{B(q-1)} & \\ & \;+\frac{b^{q}-1}{B(q-1)\Gamma (q-1)}\int_{0}^{b}\varphi_{2}\left(r\right)\left|F\left(r,\left(Sw\left(r\right),Tw\left(r\right)\right)\right)\right|dr] & \\ & \;\leq |w_{0}|+\frac{b}{\Delta }|w_{1}|+\Lambda \int_{0}^{b}\varphi_{1}\left(F\left(r,\left(Sw\left(r\right),Tw\left(r\right)\right)\right)\right)dr & \\ & \;+\Lambda \int_{0}^{b}\varphi_{2}\left(F\left(r,\left(Sw\left(r\right),Tw\left(r\right)\right)\right)\right)dr{∥w∥}_{\infty }. \end{array}$$

*Hence, for all* w∈S , *we have*

$$\begin{array}{cc} & {∥w∥}_{\infty }\leq \frac{|w_{0}|+\frac{b}{\Delta }|w_{1}|+\Lambda \int_{0}^{b}\varphi_{1}\left(F\left(r,\left(Sw\left(r\right),Tw\left(r\right)\right)\right)\right)dr}{1-\Lambda \int_{0}^{b}\varphi_{2}\left(F\left(r,\left(Sw\left(r\right),Tw\left(r\right)\right)\right)\right)dr}<+\infty , \end{array}$$

⇒S *is bounded. Therefore, the Leray-Schauder alternative theorem ensures us that* G *has at least one fixed-point in C*(*T*), *as required. Hence the theorem follows. By considering the generalized Lipschitz condition on nonlinearity*.

## 5 Uniqueness result

**Theorem 5.1** *Let* $\Omega =\{(t,w)\in ℛD^{2}:t\in J$ *and* |*w*| ≤ λ *for fixed* λ > 0}. *Suppose that* ℱ:Ω→ℛ *is absolutely continuous and there exist ϕ* ∈ *L*^1^(*J*, [0, ∞)). If ℱ(0,w(0))=0, *then* (1.1) *has a unique solution in C*(*T*), *provided*

$$\begin{array}{cc} & \Lambda \int_{0}^{b}\phi (F(r,Sw(r),Tw(r)))dr<1. \end{array}$$ (5.1)

***Proof*.** *The operator* G:C(T)→C(T) *is defined in* (4.1). *Let us first denote M* = ${\text{sup}}_{t\in J}|ℱ(t,0)|<\infty$ *and fix a real number ρ*, 0 < *ρ* ≤ λ, *s.t*.,

$$\begin{array}{cc} & \frac{|w_{0}|+\frac{b}{\Delta }|w_{1}|+M\tilde{\Lambda }}{1-\Lambda \int_{0}^{b}\phi \left(F\left(r,Sw\left(r\right),Tw\left(r\right)\right)\right)dr}\leq \rho . \end{array}$$

*We now show that* $GB_{\rho }\subset B_{\rho }$ , *where* $B_{\rho }=\left\{w\in C(T):\|w{\|}_{\infty }\leq \rho \right\}$. *By (H2), for all* $w\in B_{r}$, *one has*

$$\begin{array}{ccc} & \left|\left(Gw\right)\left(t\right)\right|\leq |w_{0}|+\frac{1}{\Delta }[|w_{1}|+\int_{0}^{b}\left(\phi \left(F\left(r,Sw\left(r\right),Tw\left(r\right)\right)\right)|F\left(r,Sw\left(r\right),Tw\left(r\right)\right)|+M\right)dr]t & \\ & \;+\frac{2-q}{B(q-1)}\int_{0}^{t}\left(\phi \left(F\left(r,Sw\left(r\right),Tw\left(r\right)\right)\right)|F\left(r,Sw\left(r\right),Tw\left(r\right)\right)|+M\right)dr & \\ & \;+\frac{1}{B(q-1)\Gamma (q-1)}\int_{0}^{t}{\left(t-r\right)}^{q-1}\left(\phi \left(F\left(r,Sw\left(r\right),Tw\left(r\right)\right)\right)|F\left(r,Sw\left(r\right),Tw\left(r\right)\right)|+M\right)dr & \\ & \;\leq |w_{0}|+\frac{1}{\Delta }[|w_{1}|+\rho \int_{0}^{b}\phi \left(F\left(r,Sw\left(r\right),Tw\left(r\right)\right)\right)dr+Mb]b & \\ & \;+\frac{2-q}{B(q-1)}[\rho \int_{0}^{b}\phi \left(F\left(r,Sw\left(r\right),Tw\left(r\right)\right)\right)dr+Mb] & \\ & \;+\frac{1}{qB(q-1)\Gamma (q-1)}b^{q-1}[q\rho \int_{0}^{b}\phi \left(F\left(r,Sw\left(r\right),Tw\left(r\right)\right)\right)dr+Mb]\;for\;allt\in J. \end{array}$$

*Therefore, for all* $w\in B_{\rho }$ , *we deduce that*

$$\begin{array}{ccc} & {∥Gw∥}_{\infty }\leq |w_{0}|+\frac{b}{\Delta }|w_{1}|+M\tilde{\Lambda }+\Lambda \rho \int_{0}^{b}\phi \left(F\left(r,Sw\left(r\right),Tw\left(r\right)\right)\right)dr & \\ & \;\leq \rho \end{array}$$

⇒G maps $B_{\rho }$.

*Next, we prove that* G *is a operator in C*(*T*). *Let* $w,\tilde{w}\in C(T)$. *By (H2), for each t* ∈ *T*, *one has*

$$\begin{array}{ccc} & |\left(Gw\right)\left(t\right)-\left(G\tilde{w}\right)\left(t\right)| & \\ & \leq \frac{t}{\Delta }\int_{0}^{b}\phi \left(F\left(r,Sw\left(r\right),Tw\left(r\right)\right)\right)\left|w\left(r\right)-\tilde{w}\left(r\right)\right|dr & \\ & +\frac{2-q}{B(q-1)}\int_{0}^{t}\phi \left(F\left(r,Sw\left(r\right),Tw\left(r\right)\right)\right)\left|w\left(r\right)-\tilde{w}\left(r\right)\right|dr & \\ & +\frac{1}{B(q-1)\Gamma (q-1)}\int_{0}^{t}{\left(t-r\right)}^{q-1}\phi \left(F\left(r,Sw\left(r\right),Tw\left(r\right)\right)\right)\left|w\left(r\right)-\tilde{w}\left(r\right)\right|dr & \\ & \leq \Lambda \int_{0}^{b}\phi \left(F\left(r,Sw\left(r\right),Tw\left(r\right)\right)\right)dr{∥w-\tilde{w}∥}_{\infty }. \end{array}$$

*Hence, for all* $w,\tilde{w}\in C(T)$ *we deduce that*

$$\begin{array}{cc} & ∥Gw-G\tilde{w}{∥}_{\infty }\leq \Lambda \int_{0}^{b}\phi \left(F\left(r,Sw\left(r\right),Tw\left(r\right)\right)\right)dr{∥w-\tilde{w}∥}_{\infty }. \end{array}$$

*The assumption* (5.1) *ensures that* G *is contractive. Hence the Banach fixed point theorem allows us to conclude that* G *has a unique fixed-point in C*(*T*), *as required. This fixed-point is the aspired solution to* (1.1).

## 6 Numerical result

This section is devoted to describe a numerical scheme based on the classical and fractional trapezoidal methods, which are used to interpolate the vector field ℱ by the first-degree polynomials [26, 41].

The fractional trapezoidal method is a numerical technique used for approximating the definite integral of a function. It is a modification of the traditional trapezoidal rule, which divides the area under a curve into trapezoids and sums their areas to estimate the integral [42–46]. It is particularly useful when dealing with functions that may have singularities or other complexities and also can provide more accurate results compared to the traditional trapezoidal rule, especially when dealing with functions that have rapidly changing behavior or singularities. It’s a simple numerical integration method that can be computationally efficient for many problems but may require more intervals to achieve high accuracy [47–49].

Now, we can directly discretize the integral equation of (1.3) to derive the numerical scheme.

Let *h* be the step size, and choose *n* + 1 equi-spaced grid points ${ℱ}_{tk}=kh:k=0,1,2,...,n$ on [0, *b*] such that 0 = *t*_0_ < *t*_1_ < *t*_2_ < … < *t*_*n*_ = *b* with some positive integers *n* and $h≔\frac{b}{n}$.

We first rewrite the integral equation of (1.3) at any point *t* = *t*_*k*_ in the following piecewise way,

$$\begin{array}{ccc} & w\left(t_{k}\right)=w_{0}+\frac{1}{\Delta }[w_{1}-\sum_{0\leq \tau \leq n-1}\int_{t_{\tau }}^{t_{\tau }+1}F\left(r,\left(Sw\left(r\right),Tw\left(r\right)\right)\right)dr]t_{k} & \\ & \;+\frac{2-q}{B(q-1)}\sum_{0\leq \tau \leq k-1}\int_{t_{\tau }}^{t_{\tau }+1}F\left(r,\left(Sw\left(r\right),Tw\left(r\right)\right)\right)dr & \\ & \;+\frac{1}{B(q-1)\Gamma (q-1)}\sum_{0\leq \tau \leq k-1}\int_{t_{\tau }}^{t_{\tau }+1}{\left(t_{k}-r\right)}^{q-1}F\left(r,\left(Sw\left(r\right),Tw\left(r\right)\right)\right)dr. \end{array}$$

In order to formulate the numerical scheme for (1.3), the nonlinear function ℱ(t,w(t)) is approximated, on each subinterval [*t*_*τ*_, *t*_*τ*_ + 1], by the following piecewise linear function,

$$\begin{array}{ccc} & F\left(t,w\left(t\right)\right)=\frac{1}{h}[\left(t_{\tau +1}-t\right)F\left(t_{\tau },\left(Sw\left(t_{\tau }\right),Tw\left(t_{\tau }\right)\right)\right) & \\ & \;+\left(t-t_{\tau }\right)F\left(t_{\tau +1}\right),\left(Sw\left(t_{\tau }+1\right),Tw\left(t_{\tau }+1\right)\right)],\;t\in \left[t_{\tau },t_{\tau }+1\right]. \end{array}$$

After integrating, one obtains the following unknown approximations

$$\begin{array}{ccc} & w\left(t_{k}\right)=w_{0} & \\ & +\frac{1}{\Delta }[w_{1}-\frac{h}{2}\sum_{0\leq \tau \leq k-1}\left[F\left(t_{\tau },\left(Sw\left(t_{\tau }\right),Tw\left(t_{\tau }\right)\right)\right)+F\left(t_{\tau +1}\right),\left(Sw\left(t_{\tau }+1\right),Tw\left(t_{\tau }+1\right)\right)\right]]t_{k} & \\ & +\frac{2-q}{B(q-1)}\frac{h}{2}\sum_{0\leq \tau \leq k-1}[F\left(t_{\tau },\left(Sw\left(t_{\tau }\right),Tw\left(t_{\tau }\right)\right)\right)+F\left(t_{\tau +1}\right),\left(Sw\left(t_{\tau }+1\right),Tw\left(t_{\tau }+1\right)\right)] & \\ & +\frac{\left(q-1\right)}{B(q-1)}\frac{h^{q}}{\Gamma (q+2)}[\left\{{\left(k-1\right)}^{q+1}-k^{q}\left(k-p-1\right)\right\}F\left(t_{0},\left(Sw\left(t_{0}\right),Tw\left(t_{0}\right)\right)\right) & \\ & +\sum_{1\leq \tau \leq k-1}\left\{{\left(k-\tau +1\right)}^{q+1}+{\left(k-\tau -1\right)}^{q+1}-2{\left(k-\tau \right)}^{q+1}\right\}F\left(t_{\tau },\left(Sw\left(t_{\tau }\right),Tw\left(t_{\tau }\right)\right)\right) & \\ & +F(t_{k},\left(Sw\left(t_{k}\right),Tw\left(t_{k}\right)\right))]. \end{array}$$ (6.1)

Due to the nonlinear nature of the function ℱ and the presence of all unknown quantities *w*(*t*_*k*_) on the RHS of each equation of (6.1), we cannot solve (6.1) for *w*(*t*_*k*_) directly, in general.

Then, the Newton-Raphson method is a numerical technique for finding approximate solutions to equations of the form *f*(*x*) = 0. It is a powerful iterative method commonly used for root finding and optimization problems.

The method starts with an initial guess and then refines that guess in each iteration until it converges to a root of the equation. It’s important to note that the Newton-Raphson method may not always converge to a solution, and it can even diverge if certain conditions are not met.

Therefore, it’s essential to choose a suitable initial guess and be aware of the characteristics of the function being analyzed. So Newton-Raphson method is used to determine each *w*(*t*_*k*_);*k* = 0, 1, 2, …, *n* from the above implicit numerical scheme (6.1). This implicit method is seldomly encountered in the numerical simulation for solving fractional differential equations. However, this method is useful in dealing with the problem (1.3).

## 7 Example

Our theoretical results are illustrated here with the example.

Let the normalization function as $ℬ(\alpha )=1-\alpha +\frac{\alpha }{\Gamma (\alpha )},\alpha \in [0,1].$ We also present the trajectory of the numerical solution to the nonlinear problem (1.1) by using the proposed numerical scheme (6.1).

{ABCDa+qw(t)=F(r,(Sw(r),Tw(r))),t∈[0,b]w(0)=w0,ABCDa+q-1w(b)=w1 (7.1)

Now, Consider the linear problem

{ABCDa+qw(t)=hλ,t∈[0,b]w(0)=w0,ABCDa+q-1w(b)=w1 (7.2)

where λ > 0 is a real number. Its exact solution is,

$$\begin{array}{cc} & w\left(t\right)=w_{0}+\frac{1}{\Delta }[w_{1}-\frac{b^{\lambda +1}}{\lambda +1}]t+\frac{2-q}{B(q-1)}\frac{t^{\lambda +1}}{\lambda +1}+\frac{q-1}{B(q-1)}\frac{\Gamma (\lambda +1)}{\Gamma (q+\lambda +1)}t^{q+\lambda } \end{array}$$

Now we consider *t* = 1, *α* = 0.5, Δ = 2, comparison of the exact and numerical solutions to Eq (6.1) for *q* = 1.5, λ = 1.5, *b* = 2, *w*_0_ = 1, *w*_1_ = 17 and *h* = 0.05.

$$\begin{array}{ccc} & w\left(t\right)=w\left(1\right)=w_{0}+\frac{1}{\Delta }[17-\frac{2^{1.5+1}}{1.5+1}]1+\frac{2-1.5}{B(1.5-1)}\frac{1^{1.5+1}}{1.5+1} & \\ & \;+\frac{1.5-1}{B(1.5-1)}\frac{\Gamma (1.5+1)}{\Gamma (1.5+1.5+1)}1^{1.5+1.5} & \\ & \;=1+\frac{1}{\Delta }\left[12.2288\right]+0.2557+5.0991 & \\ & \;=12.4692. \end{array}$$

Hence, *t* = 1; *w*(*t*) = 12.4692.

The numerical solution for *t* = 1 has been derived above. The derivation of numerical solutions *w*(*t*) for *t* ranging from 0 to 2 is similar and the values are presented in Table 1 below:

**Table 1:** The approximation values of w(t).

Numerical Values					
**t**	0	0.5	1	1.5	2
**w(t)**	1.0000	4.7398	12.4692	28.0858	55.4683

**Fig 1:**
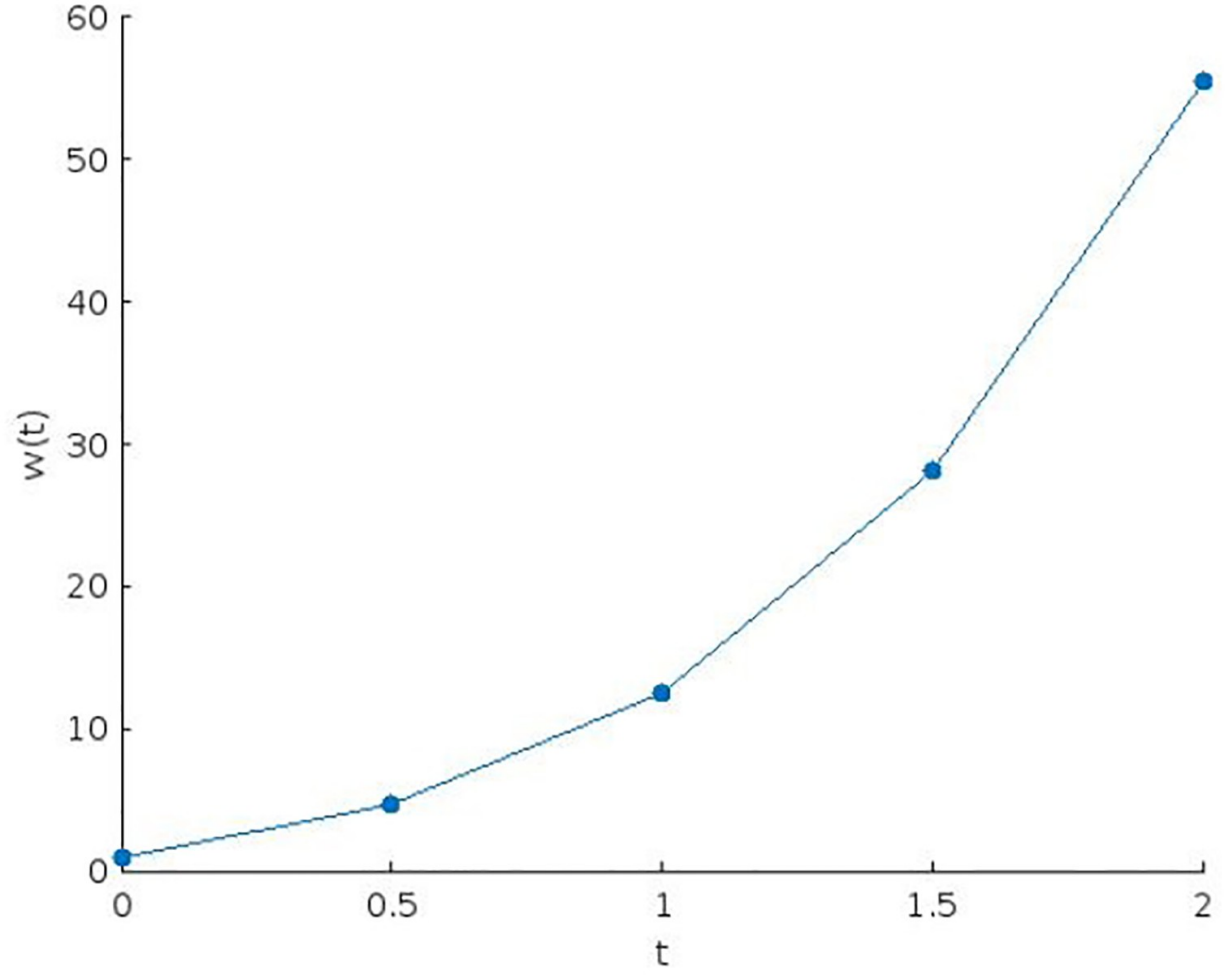
Graph of the approximate solution w(t).

The obtained results are demonstrated as a graph in Fig 1. The numerical solution shows agreement with the solution in the entire interval.

## 8 Conclusion

In this paper, the essential outcomes of the existence and uniqueness solutions are gained by using the Leray-Schauder alternative theorem and Banach fixed point theorem respectively. We also discussed a numerical scheme for (1.1), which helps to study the numerical solution when ℱ is nonlinear. Our theoretical and numerical findings were verified with examples. We have presented some important properties of higher-order AB operators. However, the stability analysis of the solution may be considered as an extension work for the problem (1.1).
